# Family members’ perception of their needs in critical care units at a tertiary hospital in Malawi: A qualitative study

**DOI:** 10.1186/s12912-023-01433-3

**Published:** 2023-08-21

**Authors:** Angellina Mankhamba Kalolo, Chimwemwe Mula, Rodwell Gundo

**Affiliations:** 1 Kamuzu University of Health Sciences (KUHeS), P/Bag 1, Lilongwe, Malawi; 2 Daeyang University (DU) College of Nursing and Midwifery, P.O. Box 30330, Lilongwe, Malawi

**Keywords:** Intensive care units, Family perceptions, Family needs, Hospitals, Malawi

## Abstract

**Background:**

Family members experience an emotional crisis when their loved one is critically ill and admitted to a critical care unit (CCU). An extensive literature has explored optimal ways to interact with families in the critical care setting, including intervention studies. What is less explored are perceptions of family members in low-income settings including Malawi. In such settings, perceptions may differ as a consequence of different cultural practices and resource limitations (personnel and technology). Therefore, this study explored family members’ perceptions of their needs in CCUs at a tertiary hospital in Malawi.

**Study designs and methods:**

The study used a qualitative descriptive design. Data were gathered through interviews with 12 participants who were purposively selected from immediate family members of patients hospitalized for 48 h or more in adult intensive care unit (ICU) and high dependency unit (HDU). The interviews were audio recorded and transcribed verbatim. Data analysis followed the steps of content analysis.

**Findings:**

The following four themes were identified: perceived information, physical, and psychosocial needs, and coping mechanisms of the family members. The family members needed information about their patient’s progress frequently and viewed this as a priority compared to other needs such as comfort and food.

**Conclusion:**

This study suggests that there should be a collaborative relationship between the CCU team and family members in order to meet their needs. Findings affirm the need for health professionals to develop guidelines or standards that promote frequent discussions with CCU family members as a means to provide support and lessen anxiety.

**Supplementary Information:**

The online version contains supplementary material available at 10.1186/s12912-023-01433-3.

## Introduction

Family members’ experiences of the crisis brought by the critical illness of their loved ones in the CCU may worsen if their needs are not met by the health personnel. A CCU is an umbrella term for an intensive care unit (ICU) and high dependency unit (HDU). The family members of patients admitted to these units experience different forms of psychological distress which include depression, anxiety and stress [1]. Nurses and doctors need to be aware of family members’ needs that are associated with hospitalization of a critically ill relative [2]. However, attention to family members is often overlooked in the healthcare environment as families are not given high priority.

Healthcare organizations consider patient and family satisfaction to be a quality indicator when assessing health services [3]. When assessing the quality of patient care in healthcare systems, these perceived needs and satisfaction with patient care are crucial [4]. Therefore, healthcare providers should aim at addressing healthcare beneficiaries’ needs through enhanced healthcare delivery. This provides a reflection of the quality of critical care provided by them especially at tertiary hospitals, hence the need to satisfy the family members’ needs. This is consistent with findings of a study by Jensen et al. [4] who reported that satisfaction of family members is of great importance in evaluating the quality of patient care in the health systems. The healthcare professionals can know family members’ perception of their needs by interacting with them during their visit to the unit.

There are few studies in Africa that reported about family members’ perception of their needs when their patient is admitted in a CCU. A study by Kohi et al. [5] in Tanzania reported that perceived needs and level of satisfaction of family members of critically ill patients call for nurses to improve the quality of care to patients’ family members, which will in return enhance the patient’s recovery. Another study done in Ethiopia by Kehali et al. [6] on lived experiences of families of patients in the ICU, pointed out that from admission till patient stabilization, families need more information and proximity to their patient than later times.

In Malawi, a quantitative study by Gundo et al. [7] compared nurses’ and families’ perception of family needs in CCU. The findings showed that family members rated high the need “to feel that the hospital personnel cares about the patient” while nurses perceived it as not a priority. Nurses ranked assurance as the highest priority need and proximity being the least. Furthermore, the findings highlighted the need for health professionals to explore and understand the concept of “caring” to meet the priority needs. Therefore, this study aimed at exploring the family members’ perceptions of their needs in the CCUs at a referral hospital in Malawi.

## Methods

### Study approach and design

This study utilized a qualitative descriptive design. The design is underpinned by the assumptions of naturalism or constructivism in which reality is not a fixed entity but rather a construction of the individuals participating in a study [8].

### Study setting

The study was conducted in adult ICU and HDU at a tertiary hospital in Malawi. The hospital is one of the public tertiary hospitals and is located in the country’s central region. It is also a teaching hospital for different health professionals including nurses and midwives, clinicians, doctors, and other allied health professionals. The hospital has one general ICU, with a capacity of eight beds. However, only five beds are mostly in use because of limited resources. The nurse: patient ratio is 1 to 2 and sometimes 1 to 1 depending on availability of nurses on duty or shift. Adjacent to the ICU, there is a surgical step down unit, an HDU with 10 beds. In both units, one or two family members per patient are allowed to visit their patients three times in a day as follows: 7:00 to 7:30am; 12:00 and 1:00 pm; and 5:00 to 6:00 pm. Scheduled family conferences are not held to share information. Physicians, nursing staff and the clinical anaesthesiologists are responsible for providing updates to the family members. In addition to the ICU located at the study setting, there are four ICUs in other public hospitals and two ICUs in private hospitals which charge user fees [7].

### Participants

Purposeful sampling technique was used to select 12 immediate family members of patients hospitalized in the CCUs. The participants included family members from the ICU (n = 8) and the HDU (n = 4). There were eight women and four males aged 35 to 64 years. Half of the participants (n = 6) dropped out of primary school, while the other half went as far as secondary and higher education. In Malawi, the terms ‘immediate family member’ and ‘guardian’ are used interchangeably when referring to a next of kin of the patient during his or her admission to the CCU [9]. The inclusion criterion was an immediate family member (spouse, father, mother, sister or brother, and child older than 18 years of age). Relatives who were not immediate family members and aged below 18 years were excluded from the study.

### Data collection

Data was gathered through face-to-face in-depth interviews using a semi-structured interview guide. Each interview was audio-recorded and notes were taken to ensure that no piece of information was missed. Preventive measures for COVID-19 were followed during the data collection exercise which included hand washing with soap, use of face masks, and social distancing of one meter between the researcher and participant. In addition, shaking hands was avoided. An interview guide was developed for this study by the primary author (see supplementary file) and was reviewed by a language and communication expert, second and third authors. Interviews were conducted in a closed room (average length 45 min) within hospital premises. The interviews were transcribed verbatim. The interviews were conducted in both Chichewa (for participants who were not comfortable with English) and English (for participants who were proficient in the language). Translation was done by a bilingual expert and was checked by an expert in language and communication. The transcripts were checked for accuracy by re-listening to the audio and comparing them with the transcripts. The trustworthiness of the findings was ensured by principles of credibility, dependability, confirmability and transferability.

### Data analysis

The data was analyzed manually utilizing the steps of content analysis as proposed by Hsieh and Shannon [10]. The steps for the analysis were as follows: preparation, organizing derived codes, making notes, labeling for codes, emergent categories, cluster formation, definitions development, and reporting the findings. The analysis was done simultaneously with data collection until data saturation was achieved. Coding was conducted by the primary author and reviewed by the second while the third author was engaged to resolve any differences.

### Ethical consideration

The proposed study was approved by the College of Medicine Research and Ethics Committee (COMREC) of the University of Malawi, reference number P.10/20/3168. Permission was also sought from the hospital’s Research Committee. The study respected the human rights of family members and patients hospitalized in the CCUs. The researcher obtained both verbal and written consent from the participants. In order to ensure anonymity, the participants were identified with numbers and all information concerning the data was kept confidential, in a locker accessible to the researcher only.

## Findings

Four themes were identified as follows: perceived information needs, perceived psychosocial needs, perceived physical needs and coping mechanisms.

### Perceived information needs

Participants expressed the need for information on the patient’s progress. They explained that they became anxious and depressed when they were not updated on their patient’s progress. Therefore, they would like to be updated on their patient’s management and condition, as stated by the following participants:

*“If healthcare workers were able to update me on the condition of my patient at least my anxiety levels would improve. But hearing nothing from those taking care of my patient is depressing. You wonder what is happening to the patient.”* (P#8- ICU).

*“I need to be updated on the progress of the patient regarding management and how he is fairing. Feedback is important to us because when I come to visit, doctors just say come and see your patient. The doctors don’t provide us with a patient’s progress report, so we just make our own judgment.”* (P#6- ICU).

Similarly, another participant said that they need adequate information so that they are able to explain to other family members:

*“I would like to hear the information from the medical staff about the condition of my patient. They should take time to explain to me, in that way that I should be able to explain to other relations at home.”* (P- 5. HDU).

### Perceived psychosocial needs

Psychosocial need is any need that is essential for mental health. Participants expressed the need for psychological support, reassurance, hope, approachable health workers, staff identity, and proximity to the patients. Participants reported that they need support from other members including relatives,

*“I need to be supported in many ways. As of now, I have just called my uncle in the village, who has come and be with me here (at the hospital). …he is the one holding my hand in support when I visit my critically ill brother.”*(P#10- ICU).

With regards to hope, participants expected health professionals to provide encouraging words or explanations that would give hope as expressed by the following participants:

*“…just an explanation or a word of giving hope or just to say we are trying but we just put everything in God’s hands, you know that will do a lot to my thinking.”* (P#3- ICU).

*“…the way I see things is that hope comes when the medical staff responds to my questions well.”* (P#4- ICU).

The participants also indicated that they needed healthcare workers who were positive, friendly, and capable of assisting them whenever they required clarification or specific information about their critically ill patients:

*“…I can say that they should be friendly so that maybe we should not be afraid eeeeh…. because sometimes we may want to ask something but because of the unfriendly manner of addressing us, we get afraid, we fail to ask them questions; we just keep quiet.”* (P#9- HDU).

In addition, some participants were concerned about the lack of health workers’ identities. They wanted the health professionals to have name tags or introduce themselves to the family members:

*“It is very difficult to tell whether it’s the nursing officer or just an ordinary worker. But all I can say is that one of the workers talk to me”* (P#11- ICU).

Furthermore, family members expressed the need to visit their patients frequently. The participants observed that frequent visits can help to allay their anxiety. This was reported one participant as follows:

*“I would love to see my brother as frequently as possible just to get some fears out of my mind. Even though I come here (*a*t the hospital) and stay for hours, I spend my day outside; they don’t allow us inside. This gives me some worries.”* (P#5- HDU).

Similarly, another participant suggested that the visiting protocol be reviewed:

*“…the members of staff should meet to review the ICU protocol especially the visiting guidelines. At least for once, they should be allowing important and closest family members to get into the ICU and see their patients. ”* (P#11-ICU).

### Perceived physical needs

Physical needs are resources that family members required for their physical health during the admission of a relative. The family members mentioned the need for shelter, financial support, and food supply because the hospital only provides for patients, and not family members or guardians.

*“…the (basic) supplies like relish and maize flour that I brought from home are finished. I don’t know what to do. I also came with my husband’s nephew (I have to provide for his needs too). It is tough for us to survive. I am so much worried.”* (P#2-HDU).

The participants also identified the need for descent shelter. Shelter was described as a room where only family members of critically ill patients could rest and discuss their experiences,

*“We know this is one of the biggest hospitals in Malawi and we feel they can consider us a room, where we can be putting up as guardians of patients in ICU”.* (P#11-ICU).

Similarly, another participant said,

*“…when they chase us from the ICU, I turn out to be a destitute as I don’t have anywhere to go. So what I do is to go outside the gate and stay there, waiting for evening staff to come and help us with a place where we can stay overnight. The workers expect us to go to the kitchen (where there is* a *guardian shelter) but the kitchen environment is not conducive at all; the kitchen surrounding is untidy and heavily congested at night.”* (P# 9- HDU).

Some participants were also concerned about financial constraints. They complained about financial costs for some services. For instance, some patients required Computed Tomography (CT) scans for diagnostic investigations which could not be done at the hospital due to nonfunctional equipment. As such the patient was referred to private hospitals for the investigations which were paid for by the family members. This was reported by one of the participants as follows,

*“I am still worried because the doctor recommended that my patient should go for scanning at a private facility, where I am supposed to pay for such (CT Scans) services. As I indicated, I am very much worried about where I would get the money from.”* (P#7-ICU).

### Coping mechanisms

Coping mechanisms are strategies used by family members to adapt to the involvement of having a relative admitted to the unit. Participants identified prayer and acceptance of the patient’s condition as their coping mechanisms.

Family members’ spiritual belief in God was critical in helping them cope with stressful events and bringing consolation into their lives. Belief in prayer gave members of the family hope or expectations. During the visiting period, some participants prayed for God’s intervention on behalf of their patients as expressed by the following participant,

*“I am a spiritual person and at the same time a guardian to …, I take some time praying for him. I can get into the room and pray silently without disturbing other patients in the ICU…. As it is now, I just commit everything in the hands of God to intervene in our situation.”* (P#1-ICU).

Furthermore, another participant said,

*“…So, all we ask for is that God should continue using the medical staff to treat and care for our patient and hoping that one day God will descend his healing hand upon our patient. But if anything happens to our patient, we know it’s the will of God.”* (P#12-ICU).

On the other hand, some participants observed that the patient’s condition was hopeless therefore they simply accepted it. This was expressed by the participants as follows,

*“… But I have just accepted the situation; I know that my child is admitted to that unit for special treatment.”* (P#1-ICU).

*“As it is at the moment, we will welcome anything that will come on our way; to be honest our patient’s condition is worrisome… So, I just accept it and go by their conditions.”* (P#5-HDU).

Participants expressed information, psychosocial and physical needs during the admission of a relative to the unit. In hopeless situations, the participants resorted to prayer and acceptance of the situation as their coping mechanisms.

## Discussion

The aim of this study was to explore the family members’ perceptions of their needs in CCUs at a referral hospital in Malawi. The major findings of this study include perceived information needs, perceived psychosocial needs, perceived physical needs, and coping mechanisms. The findings regarding family members’ anxiety, depression and stress as well as their need for timely updates in regard to the clinical condition of their family member are similar to those reported in studies of family members whose critically ill relative was admitted to a CCU in a highly developed country, suggesting these responses and needs are universal. [11].

Our sample included more female participants, as opposed to male participants, because in this setting women are most commonly those who provide care for sick family members and the individual accompanying the patient. Similarly, Gundo et al. [9] reported that women are typically considered as the family’s primary care givers in the African context. Likewise, Hoffman et al. [12] found that most of family members (hospital guardians) who cared for the sick relations were women in Malawian setting. Furthermore, Hashim et al. [13] observed that majority of the participants in their study were female care givers. It is worth noting that, some of the participants in this study were primary school dropouts. This had an implication on their understanding of their patient’s condition and their needs. According to Bandari et al. [14], educational level of patients’ family members and others is associated with their knowledge and understanding of concerns related to CCU hospitalization.

### Perceived information needs

The participants needed information about their patient’s progress and the type of care their patients received in CCU. These findings are corroborated by Abdel-Aziz et al. [15] who found that the majority of family members required more information about the patient’s diagnosis and prognosis. Furthermore, the majority of families believed that medical staff missed occasions to share information with them, or explain the care processes [14]. Similarly, Nolen et al. [16] reported that family members in their study did not always get to talk with the doctor or that they had to wait a long time to get the results of a test or operation. The explanation of what is going on instills confidence in the participants and allows them to deal with the situation better [17]. The findings support calls for health professionals to address information needs of family members because they are advocates for patients who are unable to make decisions about their care due to their critical state [13].

### Perceived psychosocial needs

Psychosocial needs comprised support, reassurance, hope, approachable health workers, staff identity, and proximity. Family members with a critically ill patient in a CCU experience high levels of anxiety, depression and stress as symptoms of psychological distress that negatively impact both the patient and family members [1]. Similarly, Gil-Juliá et al. [11] reported that almost all relatives with a loved one in CCU experienced stress. In this study, participants valued the support from health care workers and other people including relatives. This result is not surprising because the Malawian culture strengthens social cohesiveness during critical illness or grief when a family member dies [7]. Similarly, De Beer et al. [18] observed that togetherness and reassurance help to relieve the stress that is experienced by the family members. According to Adams et al. [17] nurses in CCU were positioned uniquely to provide such support because they have the most contact with both patient and family.

In addition, reassurance, which is a psychological need for family members, was identified as a critical strategy in managing their stressed mood in the CCU. Reassurance simply means the action of removing someone’s doubts or fears [19]. When family members are reassured by healthcare professionals, they gain trust and are relieved of stress. However, a study by Shorofi et al. [20] noted that the nurses and other healthcare personnel did not give reassurance to the family members, which led to some unfounded fears and uncertainty about how well the patient was responding to treatment. This is in line with the findings of a study by De Beer et al. [18] who reported that family members’ watchful attendance revealed the need for reassurance as well as the need to be close to the patient to create confidence and trust in the healthcare professionals’ treatment.

Furthermore, participants in the study were unable to distinguish healthcare workers because of the absence of their identities. This created unnecessary anxieties among immediate family members as they required an identifier to know the healthcare workers. Nurses are a significant source of information about the CCU environment, therapies, and the patient’s health state [17]. This suggests that if the participants are able to identify nurses, they may be able to approach them for support.

Furthermore, the findings showed that the participants desired frequent visits from their relatives, indicating a demand for proximity. Proximity is important to many family members because of the need to track the care that was being delivered [18]. According to Abdul Halain et al. [1], open visiting hours help to decrease anxiety in families since family members may spend more time with the patients and feel safer by being close to the patients than not being there. Similarly, Munyiginya et al. [21] reported that family members required flexible visiting hours so that they are able to visit at any time.

### Perceived physical needs

Special shelter, food supply, and financial resources were among the physical needs identified by the participants. The findings of this study revealed that there is a need for a decent shelter for family members waiting for critically ill loved ones, where they can relax and relieve tension. Consistent with the findings, Hsieh et al. [10] observed that participants in ICU expressed a desire for a better physical atmosphere in the waiting rooms. In Malawian hospitals, including where the study was conducted, at least one family member is permitted to remain in the hospital as a guardian. In most cases, their patients are referred from district (middle level) hospitals which are far from this facility, therefore they require shelter and food to meet their physiological demands.

### Coping mechanisms of family members

Prayer, acceptance of the situation, and hope were some of the coping mechanisms employed by the participants in this study. Nearly half of the participants said they believed in prayer for the betterment of their loved ones in the CCU. This is consistent with the findings of Shorofi et al. [20], who found that religious-spirituality views were particularly essential in stressful situations such as a patient’s admission to the ICU. Most people in Malawi are Christians, and praying for their relatives gives them hope and strength, believing that their relative admitted in the CCU would get the desired care and recover quickly [22].

This study found that acceptance of the situation was one of the internal motivations that propelled the participants to adapt and move forward, while their patient was admitted to the CCU. According to Shorofi et al. [20], family members change their routine organizational and personal life by adjusting to hospital routines. However, Gundo et al. [7] observed that it takes longer for a patient’s family to come to terms with the serious diagnosis. This was because telling the truth was difficult for a variety of reasons, including cultural taboos against talking about death. Provision of information to family members about their patient’s condition helps them to understand better and acknowledge the care.

## Conclusion

The findings of the study identified perceived information needs, perceived psychosocial needs, perceived physical needs, and coping mechanisms employed by the family members in CCUs in a resource-limited setting. The clinical practice implications include the need for deliberate efforts by health care providers to engage family members and address their needs in the CCUs. The findings affirm the need for health professionals to develop guidelines or standards that promote frequent discussions with family members to provide them with support and lessen their anxiety.

### Limitations

The interviews were conducted within the hospital premises whereby the participants might have been disturbed emotionally. The COVID-19 pandemic was a constraint due to restrictions which were placed on admission of the patients to the units. Data collection occurred in a public tertiary facility that serves as a teaching hospital therefore findings may not be representative of other public facilities or private facilities that do not share these characteristics. Unequal gender representation may have altered findings as perceptions of women might be different from men.

### Electronic supplementary material

Below is the link to the electronic supplementary material.

Supplementary Material 1

## Data Availability

The datasets used for the current study are available from the corresponding author upon request.

## Abbreviations

CCU: Critical Care Unit
CoM: College of Medicine
COMREC: College of Medicine Research Ethics Committee
KUHES: Kamuzu University of Health Sciences
HCS: Health Care System
HDU: High Dependency Unit
ICU: Intensive Care Unit
KCH: Kamuzu Central Hospital
KCN: Kamuzu College of Nursing
