# Supplementary material for: Family members’ perception of their needs in critical care units at a tertiary hospital in Malawi: A qualitative study
Source: BMC Nurs. 2023 Aug 21;22:274. doi: 10.1186/s12912-023-01433-3 (PMC10440910; doi:10.1186/s12912-023-01433-3)
Supplement: Supplementary file 1 — Supplementary Material 1 [file 12912_2023_1433_MOESM1_ESM.pdf]

<u>Interview guide</u>

Title of the study: **Family members' perception of their needs in critical care unit at a tertiary hospital in Malawi: A qualitative study.**

Participant code number:

Date of the interview:

General question

Can you describe your experience as family member in CCU?

Information needs of family members of patients admitted to CCU.

a) Can you explain how you received information about your family members' care in the CCU?

b) What information was provided to you about the relative admitted to the CCU?

c) Can you explain if you understand the communication or information provided by the CCU staff?

(c) Can you describe the type of information you need as a family member of a patient admitted to the CCU?

Psychosocial needs of family members of patients admitted to the CCU.

a) Can you explain your psychological and social needs as a family member of a patient admitted to the CCU?

b) Explain how your needs were met as a patient's family member in the CCU?

c) Explain what CCU staff should do to support family members of patients admitted to the unit?

Physical needs of family members of patients in CCU

a) What are the physical needs of a family member of a patient in the CCU?

b) Explain whether your needs were met as a patient's family member in the CCU?

c) Explain what CCU staff should do to support family members of patients admitted to the unit?

How do you cope with the experience in CCU?
